# Facile and Scalable Synthesis of Robust Ni(OH)_2_ Nanoplate Arrays on NiAl Foil as Hierarchical Active Scaffold for Highly Efficient Overall Water Splitting

**DOI:** 10.1002/advs.201700084

**Published:** 2017-04-18

**Authors:** Shuai Niu, Wen‐Jie Jiang, Tang Tang, Yun Zhang, Ji‐Hui Li, Jin‐Song Hu

**Affiliations:** ^1^ College of Chemistry and Material Science Hebei Normal University Shijiazhuang 050024 China; ^2^ Key Laboratory of Molecular Nanostructure and Nanotechnology, Institute of Chemistry Chinese Academy of Science Beijing 100190 China; ^3^ University of the Chinese Academy of Sciences Beijing 100049 China

**Keywords:** electrolysis, hydrogen evolution reaction (HER), nanostructures, nickel hydroxides, oxygen evolution reaction (OER)

## Abstract

Developing highly efficient low‐cost electrocatalysts for both hydrogen evolution reaction (HER) and oxygen evolution reaction (OER) in alkaline electrolyte is essential to advance water electrolysis technology. Herein, Ni(OH)_2_ nanoplates aligned on NiAl foil (Ni(OH)_2_/NiAl) are developed by simply dealloying NiAl foil in KOH, which exhibits high electrocatalytic activity for OER with a small overpotential of 289 mV to achieve 10 mA cm^−2^ and outstanding durability with no detectable degradation during long‐term operation. Furthermore, such Ni(OH)_2_/NiAl can effectively act as an active and robust hierarchical scaffold to simply electrodeposit other catalysts with intrinsically higher activity such as NiMo and NiFe nanoparticles for highly efficient HER and OER, respectively. The prepared NiFe/Ni(OH)_2_/NiAl displays superior OER catalytic activity with overpotentials of 246, 315, and 374 mV at 10, 100, and 500 mA cm^−2^, respectively. While NiMo/Ni(OH)_2_/NiAl catalyst exhibits remarkable HER performance with a small overpotential of 78 mV to deliver 10 mA cm^−2^. Consequently, the electrolysis device composed of the above two electrocatalysts demonstrates superb water splitting performance with a cell voltage of 1.59 V at 10 mA cm^−2^. These results open up opportunities to explore and optimize low‐cost advanced catalysts for energy applications.

## 1 Introduction

Electrochemical water splitting is an efficient and promising technology for the production of high‐purity hydrogen by converting electricity, especially from renewable energy, into chemical energy. However, its practical use for mass hydrogen production is limited by the large overpotential of oxygen evolution reaction (OER) at the anode and hydrogen evolution reaction (HER) at the cathode. Low‐cost and highly active electrocatalysts are needed to decrease the energy barriers, thus enhancing the energy conversion efficiency and lowering the production cost. Anode catalysts such as IrO_2_ and RuO_2_, and cathode catalysts such as Pt/C are traditionally used to promote OER and HER, respectively.^1^ However, these catalysts suffered from low abundance and high cost, limiting the large‐scale commercialization of water electrolysis. In the past few years, the development of efficient and noble‐metal‐free OER and HER catalysts based on the earth‐abundant transition metals has received extensive research interests. For example, transition metal (Ni, Co, Mo, W, etc.) oxides/hydroxides, perovskite oxides for OER,^2^ and transition metal sulfides, nitrides, selenides, phosphides, carbides, and borides for HER have been reported.^3^ Among those non‐noble‐metal electrocatalysts for OER, Ni(OH)_2_ as a typical transition metal hydroxides has received attention because of its high activity and stability. While NiMo alloy^4^ and NiFe composites^5^ are known as the highly active electrocatalysts for OER and HER, respectively. The high OER activity of NiFe catalysts originates from a strong synergistic effect from the incorporation of Fe, although the clear structural characterization and catalytic mechanisms are not fully understood yet.^6^ NiMo alloy is one of the best nonprecious‐metal electrocatalysts for HER in alkaline electrolytes because of its appropriate binding energy to hydrogen.^7^ Although substantial progress has been achieved, the challenges still remain for the exploration of low‐cost catalysts with superior activity and stability to noble metal counterparts, especially by the facile and scalable methods.

It is known that the rational design of electrode structure is vital for improving its catalytic performance. The fabrication of 3D nanostructured electrode with high active and stability is an effective strategy due to the large exposed surface area and structural merits for efficient mass transfer and gas escaping. Moreover, 3D nanostructure allows the electrode to take the advantage of strong coupling effects between different active components. 3D nanostructured electrodes usually can be fabricated by constructing 1D or 2D building block arrays, such as nanowire arrays, nanosheet or nanoplate arrays. Taking the advantage of 2D building blocks at nanoscale such as the abundant accessible surface sites, short electron transfer pathway, and easy coupling with other active materials for advancing the performance, the fabrication of such 3D nanostructured electrodes composed of 2D nanoblocks would be a possible way to achieve low‐cost and highly active electrocatalysts for electrochemical water splitting.

In this work, a scalable and low‐cost method was developed to directly grow Ni(OH)_2_ nanoplate arrays by simply dealloying nickel–aluminum (NiAl) alloy foil in KOH. NiAl foil is low‐cost, commercially available, and physically robust. The aluminum is amphoteric and easy to be etched away in alkaline solution to form hierarchically porous structure on NiAl foils, accompanying with the in situ growth of Ni(OH)_2_ nanoplate arrays on the remaining porous NiAl scaffold. It was found that the Ni(OH)_2_/NiAl prepared by this facile route showed impressive electrocatalytic activities for OER with a small overpotential of 289 mV at 10 mA cm^−2^. Moreover, this 2D Ni(OH)_2_ nanoplate arrays could be used as an active scaffold to easily couple other more active components by diverse methods such as the electrodeposition which is a versatile and scalable process with the capability of precise control of nucleation, growth for nanomaterials via a simple and low‐cost equipment.^5^ For example, the coupled NiFe/Ni(OH)_2_/NiAl hybrid exhibited excellent OER performance with low overpotentials of 246, 315, and 374 mV to reach 10, 100, and 500 mA cm^−2^, respectively, and the outstanding durability with no appreciable degradation during 10 h test. Besides, the coupled NiMo/Ni(OH)_2_/NiAl delivered remarkbale HER performance in terms of a low overpotential of 78 mV at 10 mA cm^−2^. Therefore, the alkaline water electrolyzer constructed by the above NiMo/Ni(OH)_2_/NiAl as cathode and NiFe/Ni(OH)_2_/NiAl as anode demonstrated a small cell voltage of 1.59 V at the current density of 10 mA cm^−2^ as well as the superb long‐term durability. These interesting results may inspire the development of a variety of low‐cost and efficient electrocatalysts with 3D nanostructures for diverse applications by dealloying robust and commercially available metal alloy foils as conductive and active scaffolds.

## 2 Results and Discussion

**Figure 1:**
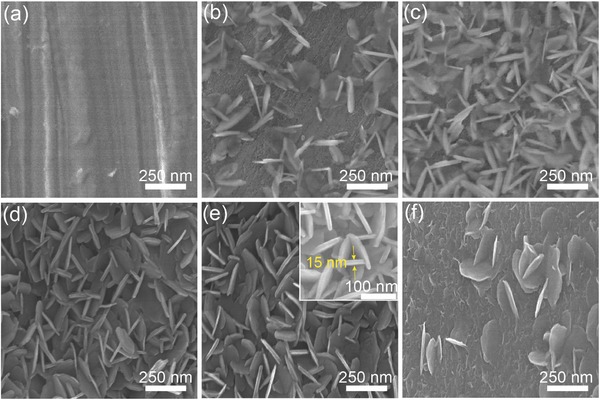
SEM images of a) as‐received NiAl foil, b) Ni(OH)_2_/NiAl‐1, c) Ni(OH)_2_/NiAl‐3, d) Ni(OH)_2_/NiAl‐5, e) Ni(OH)_2_/NiAl‐6, and f) Ni(OH)_2_/Ni‐6. The inset in panel (e) is high‐resolution SEM image.

In brief, a series of Ni(OH)_2_ nanoplates on NiAl alloy foil were synthesized simply by soaking NiAl alloy foils in 5 m KOH at 95 °C for a couple of days (see the Experimental Section for details), noted as Ni(OH)_2_/NiAl‐*n*, where *n* represents the number of the days. Their morphologies were investigated by scanning electron microscopy (SEM). The blank NiAl alloy foil had a smooth surface (**Figure** **1**a). Only Ni and Al elements were detected by energy‐dispersive X‐ray spectrum (EDS) (Figure S1a, Supporting Information). After soaking for 1 d, scattered nanoplates showed up on the surface (Figure 1b). As the soaking time increased to 3 d, the number of nanoplates on the surface increased significantly (Figure 1c) and elemental oxygen could be detected except elemental Ni and Al in EDS analysis (Figure S1b, Supporting Information), implying the possible presence of Ni(OH)_2_ given that it is the potential product of the reaction of Ni in KOH. The nanoplate density kept increasing as the reaction proceeded and covered the entire surface of NiAl (Figure 1d,e), resulting in the disappearance of Al signal in EDS spectrum of Ni(OH)_2_/NiAl‐6 (Figure S1c, Supporting Information). The high‐resolution SEM image showed that the thickness of nanoplate was approximately 15–20 nm (inset in Figure 1e). In comparison, pure Ni foil was also treated under the same condition. The nanoplates in low density were obtained (Figure 1f), indicating that the dealloying process played a significant role in the formation of such nanoplates.

**Figure 2:**
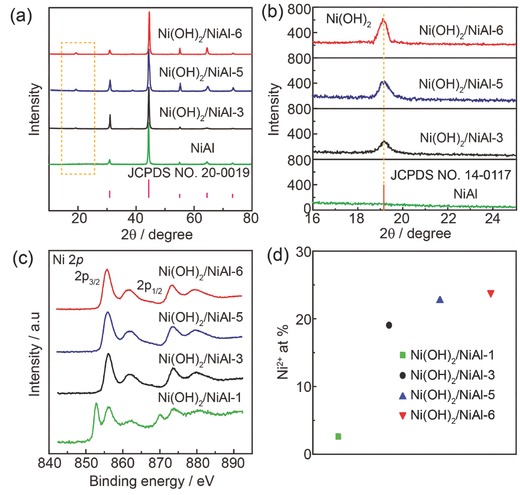
a) XRD patterns of Ni(OH)_2_/NiAl‐3, Ni(OH)_2_/NiAl‐5, Ni(OH)_2_/NiAl‐6, and blank NiAl. The purple vertical lines are the standard reference pattern of NiAl alloy (JCPDS No. 20‐0019). b) The enlarged patterns in the range of 16°–25°. The orange vertical line is the strongest line of Ni(OH)_2_ (JCPDS No. 14‐0117) pattern. c) Ni 2*p* XPS spectra and d) the content of Ni^2+^ from XPS spectra for Ni(OH)_2_/NiAl‐1, Ni(OH)_2_/NiAl‐3, Ni(OH)_2_/NiAl‐5, Ni(OH)_2_/NiAl‐6.

The structure and composition of nanoplates grown on the surface of NiAl alloy were further examined by X‐ray diffraction (XRD). In **Figure** **2**a, the blank NiAl alloy showed the strong XRD peaks at 30.9°, 44.3°, 55.0°, 64.4°, and 73.2°, which were well indexed to the diffractions of NiAl alloy (purple lines, JCPDS No. 20‐0019). Except those peaks, a peak at 19.2° in the patterns of Ni(OH)_2_/NiAl‐3, Ni(OH)_2_/NiAl‐5, Ni(OH)_2_/NiAl‐6 was clearly distinguished, which matched well with the strongest diffraction from (001) plane of Ni(OH)_2_ (JCPDS No. 14‐0117), suggesting the nanoplates were Ni(OH)_2_. The enlarged patterns in the range of 16°–25° (Figure 2b) clearly illustrated that the peak intensity increased with the increase of reaction time, which was due to the increased density of nanoplates as indicated by SEM results. Then, X‐ray photoelectron spectroscopy (XPS) was employed to analyze the chemical states of Ni in the Ni(OH)_2_/NiAl. As shown in Figure 2c, the Ni 2*p*_3/2_ peak at 852.2 and 855.8 eV of Ni(OH)_2_/NiAl‐1 were assigned to Ni^0^ and Ni^2+^, respectively.^8^ The signal of Ni^0^ came from the metallic NiAl substrate, indicating the incomplete coverage of Ni(OH)_2_ nanoplates on NiAl foil as shown in the SEM image (Figure 1b). However, only Ni^2+^ peak was observed on Ni(OH)_2_/NiAl‐3, Ni(OH)_2_/NiAl‐5, and Ni(OH)_2_/NiAl‐6. The content of Ni^2+^ on the surface of these samples was given by fitting Ni 2*p*_3/2_ spectra (Figure S2, Supporting Information). In Figure 2d, the content of Ni^2+^ is 3.6, 19.0, 23.0, and 24.0 at% for Ni(OH)_2_/NiAl‐1, Ni(OH)_2_/NiAl‐3, Ni(OH)_2_/NiAl‐5, and Ni(OH)_2_/NiAl‐6, respectively. The increased content of Ni^2+^ from Ni(OH)_2_/NiAl‐1 to Ni(OH)_2_/NiAl‐3 was attributed to the gradually increased amount of Ni(OH)_2_ nanoplates with the extended reaction time. While the small difference between Ni(OH)_2_/NiAl‐5 and Ni(OH)_2_/NiAl‐6 implied the full coverage of Ni(OH)_2_ nanoplates on NiAl foil.

**Figure 3:**
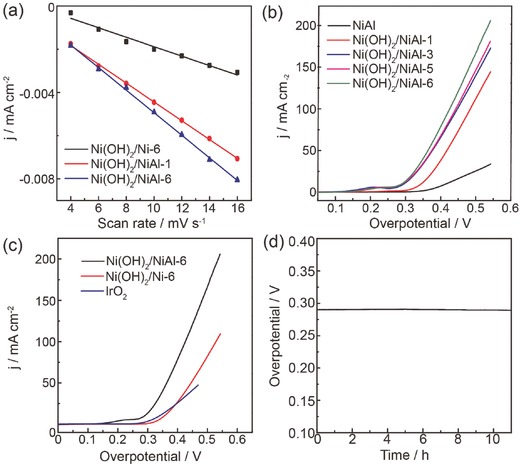
a) Current density as a function of scan rate for as‐prepared catalysts. b,c) LSV curves for as‐prepared catalysts at a scan rate of 5 mV s^−1^ for OER. d) Chronopotentiometric curve recorded on Ni(OH)_2_/NiAl‐6 at a constant current density of 10 mA cm^−2^. All curves were recorded without iR‐correction.

The electrochemically surface areas (ECSA) of the as‐prepared catalysts were determined by measuring the electrochemical double layer capacitances (*C*_dl_) in 1 m KOH, which were calculated from cyclic voltammetry measurements at different scan rates (Figure S3, Supporting Information). According to the slope of plots in **Figure** **3**a, Ni(OH)_2_ on Ni foil after 6 d soaking (Ni(OH)_2_/Ni‐6) showed a small *C*_dl_ value of 0.249 mF cm^−2^, whereas it was dramatically increased to 0.434 mF cm^−2^ for Ni(OH)_2_/NiAl‐1 and 0.522 mF cm^−2^ for Ni(OH)_2_/NiAl‐6. The highest ECSA value of Ni(OH)_2_/NiAl‐6 should be originated from the highest density of Ni(OH)_2_ nanoplates. The linear sweep voltammetry (LSV) polarization curves were recorded at a scan rate of 5 mV s^−1^ to evaluate the OER performance in terms of the overpotential at 10 mA cm^−2^ (a metric relevant to solar fuel synthesis). All the measurements were performed without iR‐correction unless specified. In Figure 3b, the blank NiAl showed the worst electrocatalytic performance with a larger overpotential of 416 mV at 10 mA cm^−2^. When Ni(OH)_2_ nanoplates were grown on its surface, this overpotential was greatly improved. For Ni(OH)_2_/NiAl‐6, the overpotential at 10 and 100 mA cm^−2^ were 289 and 425 mV, respectively, which is an outstanding catalytic OER performance among various reported monometallic Ni‐based catalysts,^9^ and commercial IrO_2_ (339 mV at 10 mA cm^−2^, Figure 3c). While pure Ni foil was used instead of NiAl alloy, Ni(OH)_2_/Ni‐6 required a large overpotential of 355 mV to reach 10 mA cm^−2^, which was attributed to the low density of Ni(OH)_2_ nanoplates. To assess the durability of the Ni(OH)_2_/NiAl‐6, chronopotentiometric measurement was conducted at a constant current density of 10 mA cm^−2^. The overpotential (without iR correction) remains almost unchanged during 10 h electrolysis (Figure 3d). Such an outstanding durability of Ni(OH)_2_/NiAl‐6 indicated the remarkable stability of Ni(OH)_2_ nanoplates grown on NiAl alloy foil via the present method. The superior activity as well as durability of Ni(OH)_2_/NiAl‐6 electrode demonstrated that the present Ni(OH)_2_/NiAl could be used as an active electrode for OER itself as well as the robust active scaffold to couple other active components to further boost the OER performance. Moreover, the electrocatalytic activity of Ni(OH)_2_/NiAl‐6 for HER was also examined in 1 m KOH and an overpotential (without iR correction) of 292 mV was required to deliver 10 mA cm^−2^ (Figure S4, Supporting Information).

**Figure 4:**
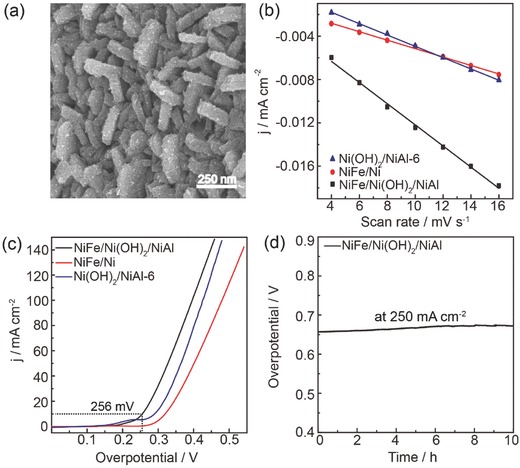
a) SEM image of NiFe/Ni(OH)_2_/NiAl. b) Current density as a function of scan rate for as‐prepared catalysts. c) OER polarization curves of as‐prepared catalysts at a scan rate of 5 mV s^−1^. d) Chronopotentiometric curve of NiFe/Ni(OH)_2_/NiAl recorded at a large constant current density of 250 mA cm^−2^. All curves were recorded without iR‐correction.

In order to further boost the OER activity of the present electrode, the NiFe composites were electrodeposited on the above Ni(OH)_2_/NiAl‐6 scaffold according to the previous report (see the Experimental Section for details).^10^ The morphology of obtained NiFe/Ni(OH)_2_/NiAl was probed by SEM as shown in **Figure** **4**a. The feature of nanoplates was well maintained while the thickness increased to 50–55 nm, indicating the successful deposition of NiFe composites on Ni(OH)_2_ nanoplates. XPS (Figure S5, Supporting Information) and EDS (Figure S6, Supporting Information) analysis evidenced the existence of metallic Ni and Fe elements. The binding energy peak of Ni 2*p*_3/2_ at 856 eV and Fe 2*p*_3/2_ at 712 eV were well consistent with the previous report, corroborating the successful deposition of NiFe.^5^ The electrodepositions of NiFe composites on Ni(OH)_2_/NiAl scaffold and blank Ni foil were optimized through varying the electrodeposition time and measuring their OER performance as shown in Figure S7 in the Supporting Information. It was found that the OER curves remained unchanged after 200 s deposition on Ni(OH)_2_/NiAl scaffold, which was used for the following experiments. The *C*_dl_ value of NiFe/Ni(OH)_2_/NiAl was 0.815 mF cm^−2^, which was much larger than 0.522 mF cm^−2^ for Ni(OH)_2_/NiAl‐6 scaffold itself (Figure 4b; Figure S8, Supporting Information). When NiFe composites were electrodeposited on blank Ni foil (NiFe/Ni), the *C*_dl_ value was only 0.272 mF cm^−2^ under the optimized deposition time of 300 s, suggesting that the presence of Ni(OH)_2_ nanoplates remarkably increased the ECSA and thus provided much more accessible catalytic sites. As expected, NiFe/Ni(OH)_2_/NiAl hybrids exhibited outstanding catalytic performance for OER with an ultrasmall overpotential of 256 mV to reach 10 mA cm^−2^ (without iR‐compensation), which is 46 and 33 mV lower than those of NiFe/Ni and Ni(OH)_2_/NiAl‐6, respectively. After the iR‐compensation, the overpotential at 10, 100, and 500 mA cm^−2^ were 246, 315, and 374 mV, respectively (Figure S9, Supporting Information). Such performance ranked as one of the most active nonprecious metal OER electrocatalysts (Table S1, Supporting Information). Importantly, to demonstrate its potential application in practical system, the durability of NiFe/Ni(OH)_2_/NiAl electrode was tested at a high current density of 250 mA cm^−2^. Figure 4d showed that the overpotential increased by only 20 mV after 10 h test.

**Figure 5:**
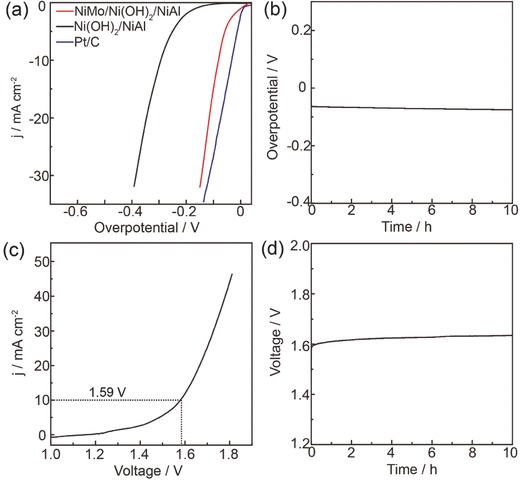
a) LSV curves for NiMo/Ni(OH)_2_/NiAl, Ni(OH)_2_/NiAl, and Pt/C at a scan rate of 5 mV s^−1^ for HER. b) Chronopotentiometric curve of NiMo/Ni(OH)_2_/NiAl at a constant current density of 10 mA cm^−2^. c) LSV curve for overall water splitting in a two‐electrode configuration, where NiFe/Ni(OH)_2_/NiAl was used as anode and NiMo/Ni(OH)_2_/NiAl as cathode. d) Chronopotentiometry curve of the overall water splitting device under a current density of 10 mA cm^−2^.

NiMo alloy has appropriate binding energy of hydrogen and thus a high activity for HER.^4^ Therefore, we simply electrodeposited NiMo alloy on our Ni(OH)_2_/NiAl scaffold to achieve a more efficient HER electrode (see the Experimental Section for details, noted as NiMo/Ni(OH)_2_/NiAl). The Ni 2*p*_3/2_ peak at 853.0 eV and Mo 3*d*_5/2_ peak at 228.1 eV were characteristic binding energy of Ni^0^ and Mo^0^ (Figure S10, Supporting Information), suggesting the successful fabrication of NiMo alloy.^4^ The electrodeposition time was controlled to optimize its ECSA value. After the electrodeposition for 3600 s, NiMo/Ni(OH)_2_/NiAl exhibited the largest *C*_dl_ value of 0.037 mF cm^−2^ and thus the best HER performance (Figure S11 and S12, Supporting Information). The HER activity of NiMo/Ni(OH)_2_/NiAl, Ni(OH)_2_/NiAl as well as commercial Pt/C was evaluated as shown in **Figure** **5**a. Compared with the overpotential of 292 mV for Ni(OH)_2_/NiAl, after electrodeposition of NiMo alloy nanoparticles, the overpotential at 10 mA cm^−2^ for NiMo/Ni(OH)_2_/NiAl strikingly decreased to 78 mV, which was approaching the Pt/C (33 mV) (Figure 5a). This performance is prominent among the reported non‐noble HER electrocatalysts (Table S2, Supporting Information).^11^ We further explored its long‐term electrochemical stability in 1 m KOH. The time‐dependent overpotential at a fixed current density of 10 mA cm^−2^ demonstrated an overpotential loss of only 15 mV after 10 h test (Figure 5b), indicating the outstanding durability. Based on the above inspiring OER and HER results, we accordingly assembled a water electrolyzer using NiMo/Ni(OH)_2_/NiAl as cathode and NiFe/Ni(OH)_2_/NiAl as anode. This electrolyzer exhibited superb performance in 1 m KOH in terms of a cell voltage of 1.59 V to deliver 10 mA cm^−2^ during the overall water splitting (Figure 5c). This small voltage is in leading position in most of reported electrocatalysts (Table S3, Supporting Information).[[qv: 9a,12]] The durability test showed that the electrode potential at 10 mA cm^−2^ slightly increased to 1.63 V after the continue operation for 10 h, suggesting its good durability (Figure 5d).

## 3 Conclusion

In conclusion, a highly active Ni(OH)_2_ nanoplate array on NiAl foil was developed through a dealloying and in situ growth route by simply soaking commercial NiAl alloy foil in alkaline solution. The time‐dependent experiments showed that the optimized Ni(OH)_2_/NiAl electrode exhibited superior OER performance with a small overpotential of 289 mV at 10 mA cm^−2^ and outstanding durability in 1 m KOH. The superb stability of Ni(OH)_2_/NiAl allowed it to effectively act as a hierarchical active scaffold to couple other component to further boost the OER and HER performance. After coupling with NiFe, the obtained NiFe/Ni(OH)_2_/NiAl exhibited an outstanding OER activity with ultrasmall overpotentials of 246, 315, and 374 mV to achieve 10, 100, and 500 mA cm^−2^, respectively. The coupled NiMo/Ni(OH)_2_/NiAl electrode displayed a remarkable HER performance with a low overpotential of 78 mV at 10 mA cm^−2^. Combining these two efficient electrodes, a high‐performance water electrolyzer demonstrated a low cell voltage of 1.59 V to achieve 10 mA cm^−2^ as well as excellent stability. In view of the simple and scalable preparation of the present electrodes, the developed strategy may inspire the exploration of low‐cost highly active multiple‐scale nanostructured electrodes consisting of transition metal hydroxides and metal oxides for diverse applications, such as electrolyzer, supercapacitor, solar cells, and fuel cells.

## Experimental Section

*Materials*: All reagents were of analytical grade and used without further purification. NiAl alloy foil was purchased from Beijing Purui Advanced Material Technology Co Ltd. KOH was purchased from Beijing Chemical Works. Nickel nitrate hexahydrate, iron nitrate nonahydrate, nickel sulfate, sodium citrate, sodium molybdate, Ni foil, and sodium chloride were obtained from Alfa Aesar. Milli‐Q water (resistance of 18.2 MΩ cm at 25 °C) were used for experiments.

*Preparation of Ni(OH)_2_/NiAl and Ni(OH)_2_/Ni electrodes*: NiAl alloy foil was ultrasonically cleaned in ethanol and acetone for 30 min prior to use. The precleaned NiAl alloy foil was soaked in 5 m KOH at 95 °C for 1, 3, 5, or 6 d, then cooled to room temperature to obtain Ni(OH)_2_/NiAl‐*n* electrodes, where *n* represents the soaking time in days. For comparison, Ni foil was also subjected to the same treatment as that for Ni(OH)_2_/NiAl.

*Preparation of NiFe/Ni(OH)_2_/NiAl and NiFe/Ni electrodes*: NiFe were electrodeposited onto the surface of Ni(OH)_2_/NiAl electrode to obtain NiFe/Ni(OH)_2_/NiAl hybrid according to the previous report.^10^ Typically, the electrodeposition was carried out in a typical three‐electrode setup, using the obtained Ni(OH)_2_/NiAl as working electrode, Pt wire as counter electrode, and Ag/AgCl (3 m KCl) as reference electrode. The electrodeposition electrolyte is composed of Fe(NO_3_)_3_·9H_2_O (3 × 10^−3^ m) and Ni(NO_3_)_2_·6H_2_O (3 × 10^−3^ m). The deposition potential was fixed at −1 V versus Ag/AgCl and electrodeposition time optimized the electrocatalytic activity of NiFe/Ni(OH)_2_/NiAl. For comparison, the NiFe were also electrodeposited on precleaned Ni foils to obtain NiFe/Ni electrode using the same electrodeposition procedure.

*Preparation of NiMo/Ni(OH)_2_/NiAl electrode*: NiMo nanoparticles were electrodeposited in a 100 mL beaker with Ni(OH)_2_/NiAl as working electrode, Ag/AgCl as reference electrode, and carbon rod as counter electrode. The deposition solution consisted of 3.52 g of sodium citrate, 1.92 g of sodium molybdate, and 3.16 g of nickel sulfate in 80 mL water with addition of 4 mL NH_3_·H_2_O. The deposition process was proceeded by fixing current density at −80 mA cm^−2^ for 60 min. Electrodeposition time was optimized the electrocatalytic activity of NiMo/Ni(OH)_2_/NiAl.

*Characterizations*: The morphology of obtained electrodes were examined on a Hitachi S‐4800 scanning electron microscope at an accelerating voltage of 15 kV. Powder XRD patterns were recorded on a Regaku D/Max‐2500 diffractometer equipped with a Cu Kα1 radiation (λ = 1.54056 Å) at a scan rate of 2° min^−1^. The surface elemental information was obtained by XPS on the Thermo Scientific ESCALab 250Xi using 200 W monochromated Al Kα radiation. The binding energies for all spectra were calibrated with respect to C 1*s* line at 284.8 eV.

*Electrochemical tests—Oxygen Evolution Reaction and Hydrogen Evolving Reaction*: All the electrochemical tests were conducted on an electrochemical workstation CHI 760E. Using a conventional three‐electrode cell in 1 m KOH electrolyte. The as‐prepared electrodes, saturated calomel electrode, and Pt wire were used as working electrode, reference electrode, and counter electrode, respectively. The LSV curves were recorded at a scan rate of 5 mV s^−1^. The long‐term durability test was performed using chronopotentiometry method at a constant current density. All curves were recorded without iR‐compensation unless specified. The *C*_dl_ values for as‐parepared working electrodes are determined from the cyclic voltammograms in the double layer region (without Faradaic processes) at different scan rates.

*Electrochemical tests—Overall Water Splitting*: Overall water splitting measurement was performed in a two‐electrode system consisting of NiFe/Ni(OH)_2_/NiAl as anode electrode and NiMo/Ni(OH)_2_/NiAl as cathode electrode. The linear sweep voltammetry curve for overall water splitting was scanned in 1 m KOH at a rate of 5 mV s^−1^.

## Supporting information

Supplementary
