# Supplementary material for: Facile and Scalable Synthesis of Robust Ni(OH)2 Nanoplate Arrays on NiAl Foil as Hierarchical Active Scaffold for Highly Efficient Overall Water Splitting
Source: Adv Sci (Weinh). 2017 Apr 18;4(8):1700084. doi: 10.1002/advs.201700084 (PMC5566344; doi:10.1002/advs.201700084)
Supplement: Supplementary file 1 — Supplementary [file ADVS-4-na-s001.pdf]

Copyright WILEY-VCH Verlag GmbH & Co. KGaA, 69469 Weinheim, Germany, 2017.

Supporting Information

for *Adv. Sci.,* DOI: 10.1002/advs.201700084

Facile and Scalable Synthesis of Robust $Ni(OH)_2$ Nanoplate Arrays on NiAl Foil as Hierarchical Active Scaffold for Highly Efficient Overall Water Splitting

Shuai Niu, Wen-Jie Jiang, Tang Tang, Yun Zhang, Ji-Hui Li, and Jin-Song Hu**

Copyright WILEY-VCH Verlag GmbH & Co. KGaA, 69469 Weinheim, Germany, 2016.

Supporting Information

Facile and Scalable Synthesis of Robust $Ni(OH)_2$ Nanoplate Arrays on NiAl Foil as Hierarchical Active Scaffold for Highly Efficient Overall Water Splitting

Shuai Niu,[ab] Wen-Jie Jiang,[ac] Tang Tang,[a] Yun Zhang,[a] Ji-Hui Li,[b] and Jin-Song Hu*[ac]*

S. Niu, [+] W.-J. Jiang, [+] T. Tang, Dr. Y. Zhang, Prof. J.-S. Hu
[a] Key Laboratory of Molecular Nanostructure and Nanotechnology, Institute of Chemistry, Chinese Academy of Science, Beijing 100190, China
E-mail: hujs@iccas.ac.cn

S. Niu, Prof. J-H. Li
[b] College of Chemistry and Material Science, Hebei Normal University, Shijiazhuang 050024 (China)
E-mail: chemlijh@163.com

W.-J. Jiang, Prof. J.-S. Hu
[c] University of the Chinese Academy of Sciences
Beijing 100049 (China)

[+] These authors contributed equally.

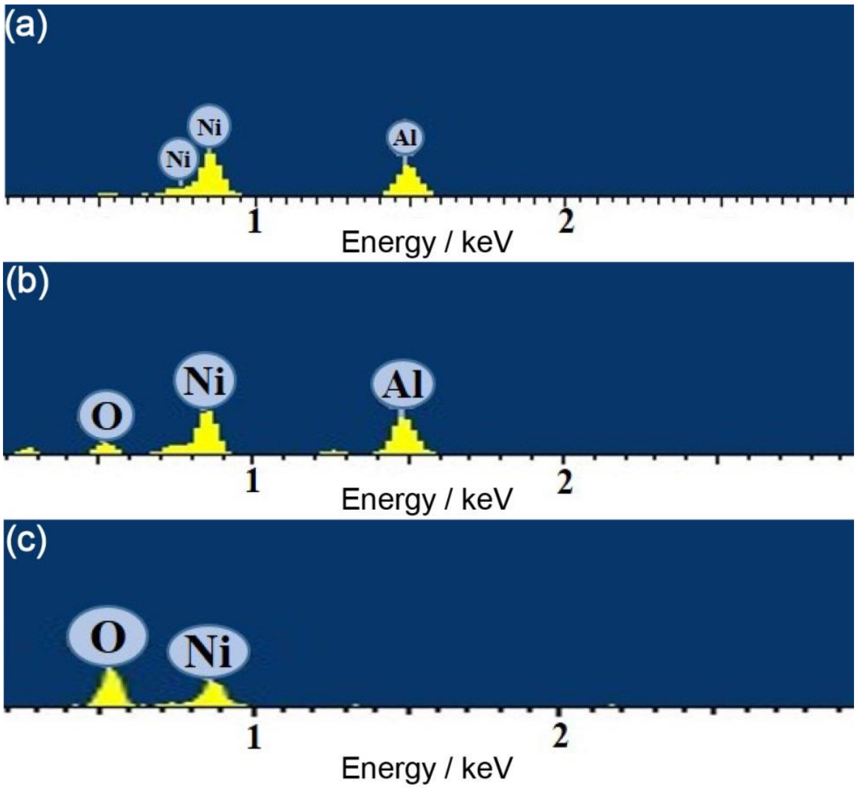

Figure S1. EDS spectra of (a) blank NiAl alloy, (b) $Ni(OH)_2$/NiAl-3, and (c) $Ni(OH)_2$/NiAl-6.

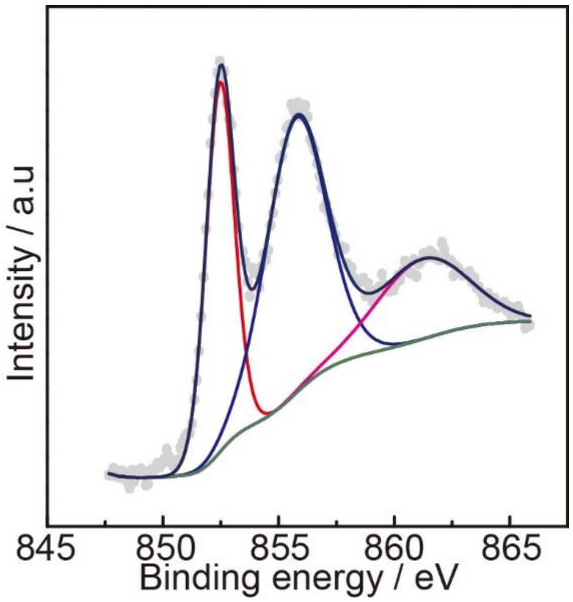

Figure S2. The deconvoluted Ni $2p_{3/2}$ XPS spectra of $Ni(OH)_2$/NiAl-1.

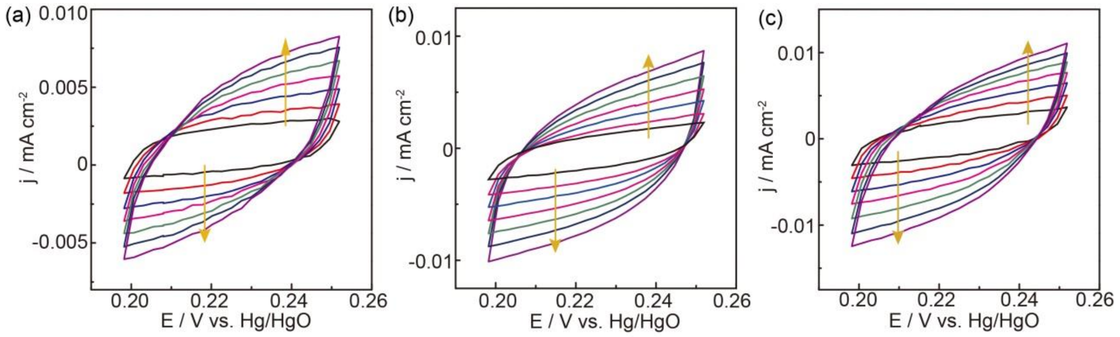

Figure S3. Cyclic voltammograms of (a) $Ni(OH)_2$/Ni-6, (b) $Ni(OH)_2$/NiAl-1, and (c) $Ni(OH)_2$/NiAl-6 in the double layer region (without Faradic process) at the scan rates of 4, 6, 8, 10, 12, 14, 16 mV/s varying along the arrow direction.

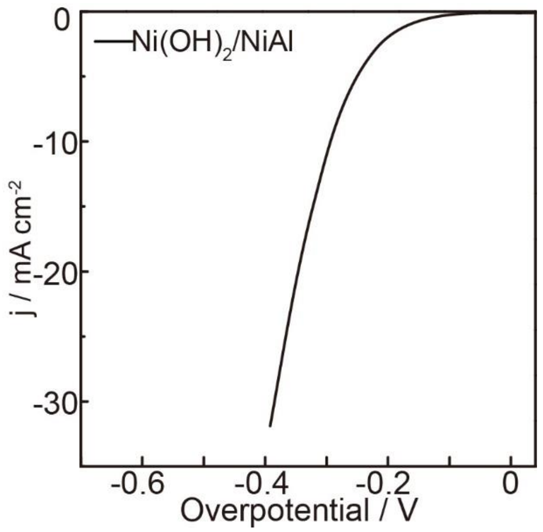

Figure S4. HER polarization curve of $Ni(OH)_2$/NiAl-6 without iR-correction.

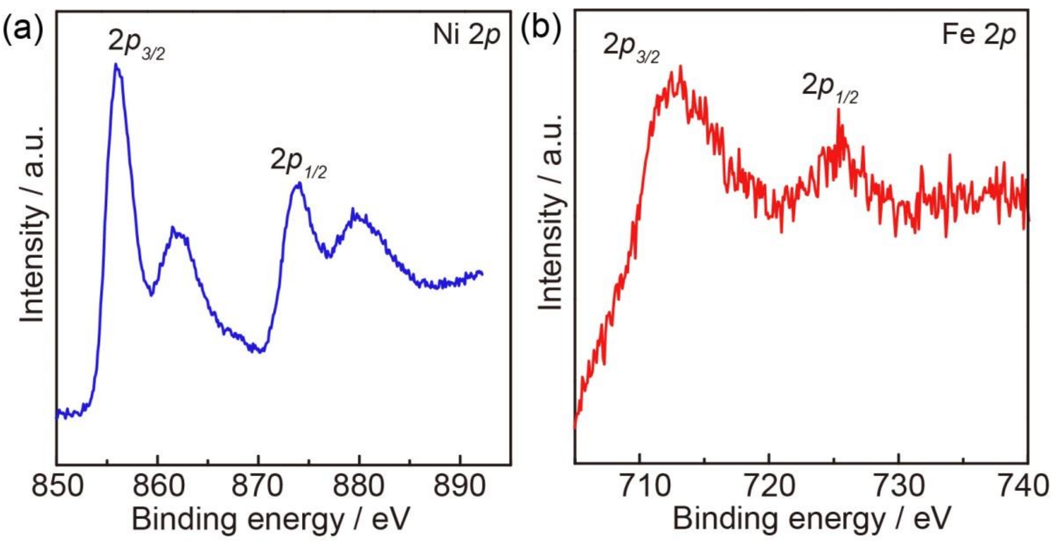

Figure S5. (a) Ni 2*p* and (b) Fe 2*p* XPS spectra of NiFe/$Ni(OH)_2$/NiAl hybrid.

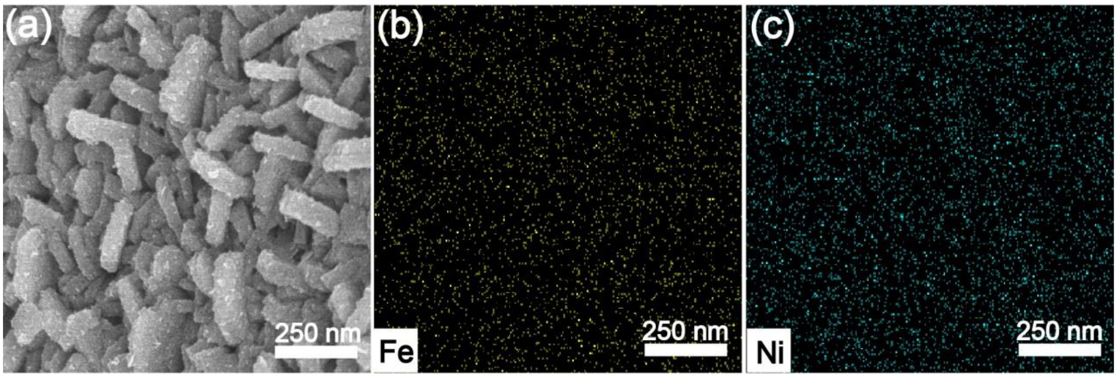

Figure S6. EDS mapping of NiFe/$Ni(OH)_2$/NiAl.

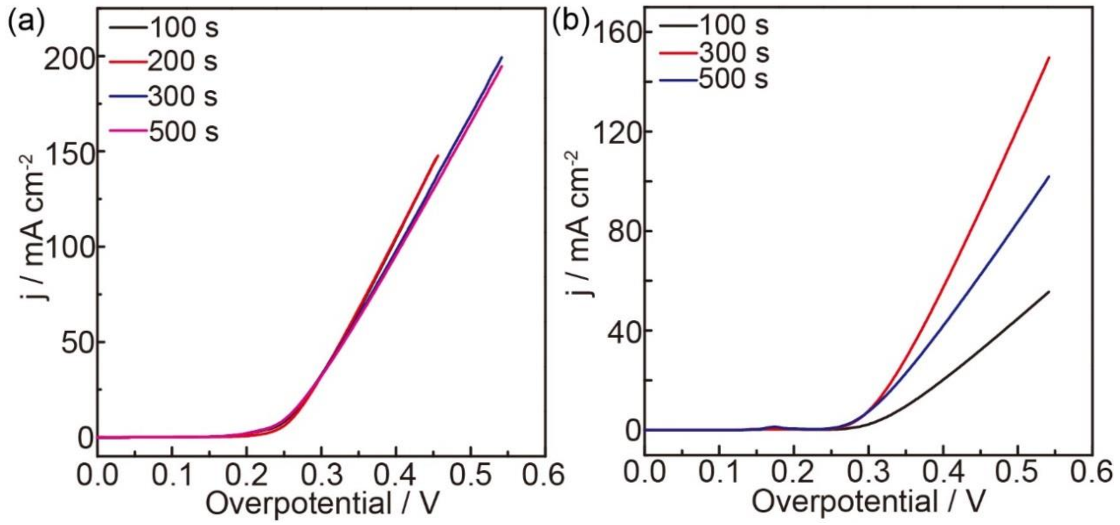

Figure S7. OER polarization curves of (a) NiFe/Ni(OH)$_2$/NiAl prepared with different electrodeposition time and (b) NiFe/Ni prepared with different electrodeposition time.

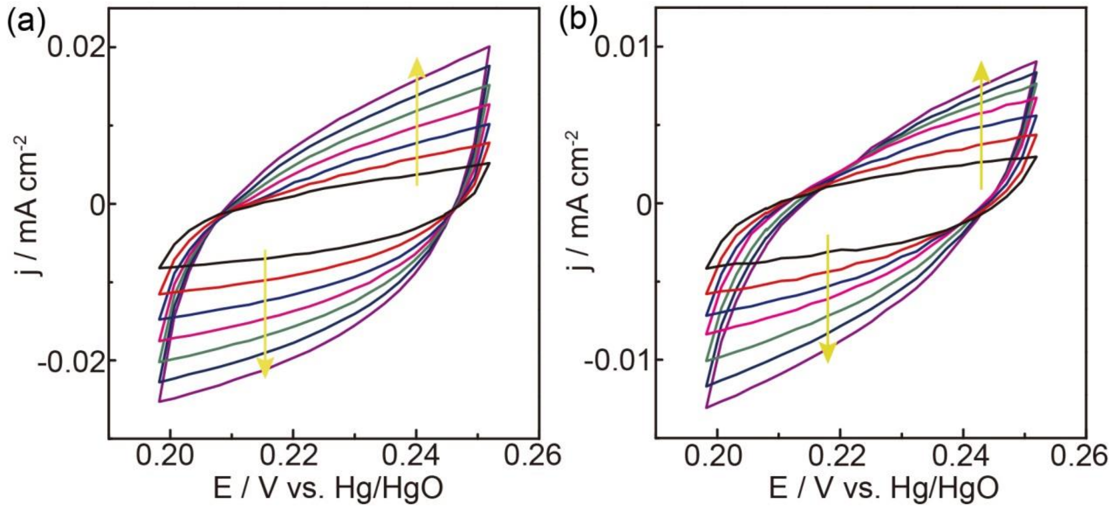

Figure S8. Cyclic voltammograms of (a) NiFe/Ni(OH)$_2$/NiAl and (b) NiFe/Ni in the double layer region (without Faradic process) at the different scan rates of 4, 6, 8, 10, 12, 14, 16 mV/s varying along the arrow direction.

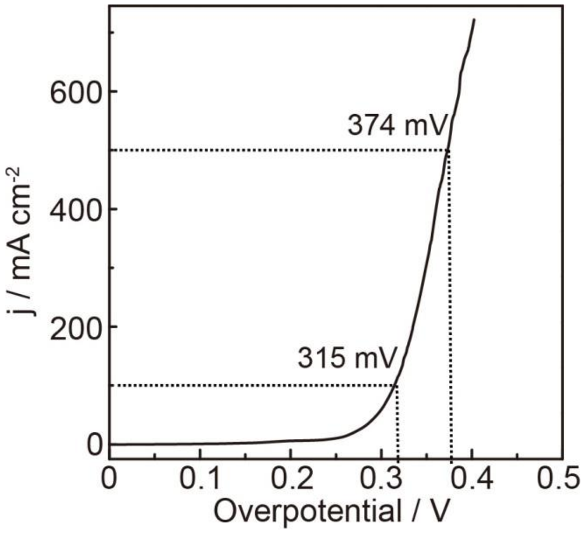

Figure S9. OER polarization curve of NiFe/Ni(OH)$_2$/NiAl with iR-correction.

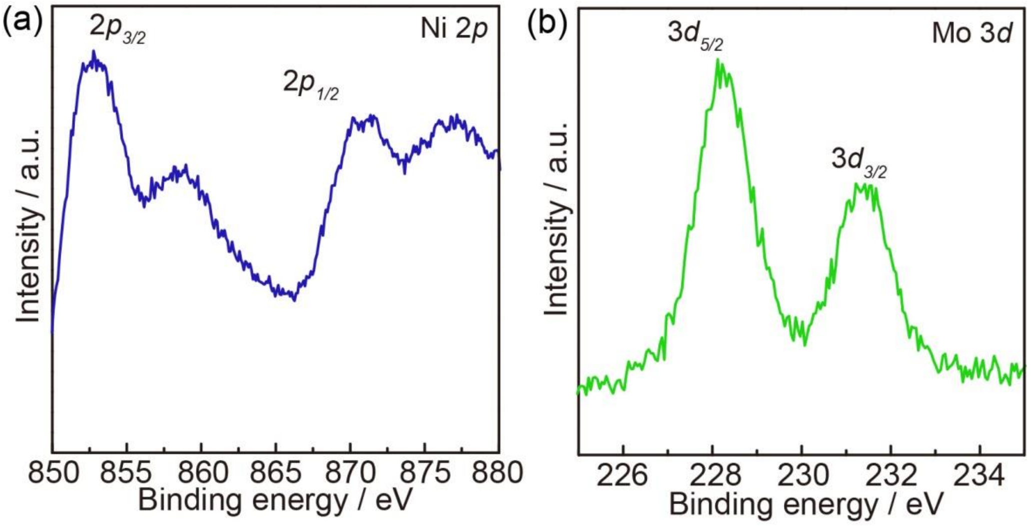

Figure S10. (a) Ni 2*p* and (b) Mo 3*d* XPS spectra of NiMo/Ni(OH)$_2$/NiAl hybrid.

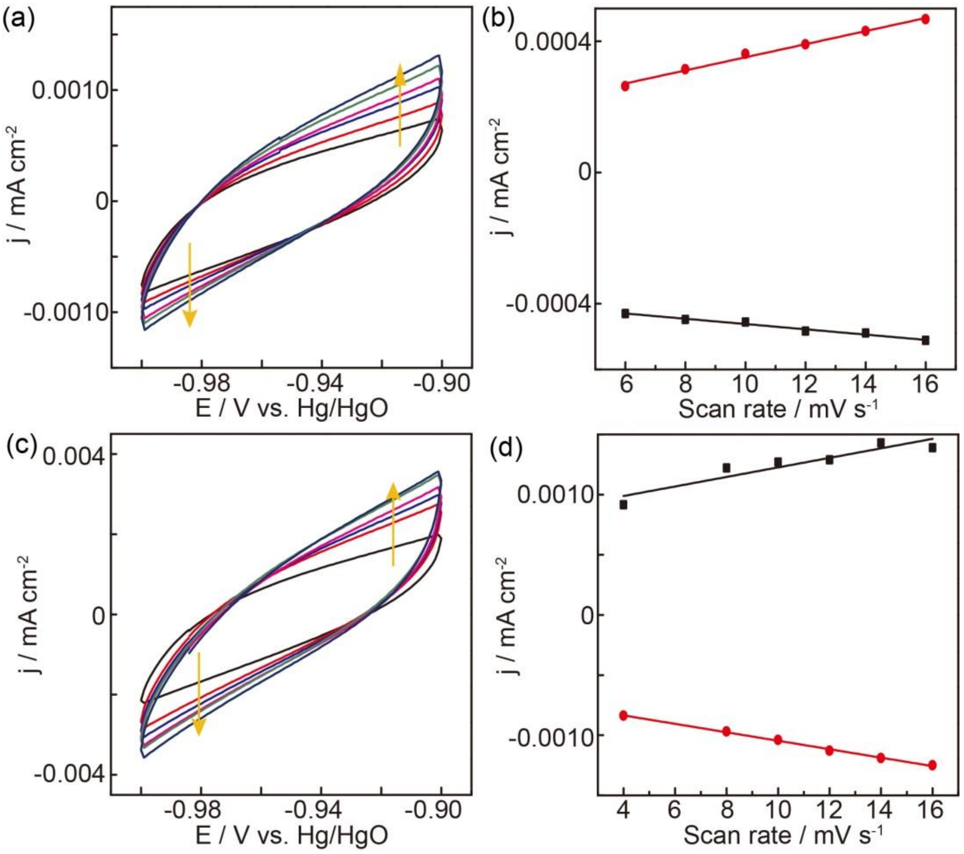

Figure S11. (a, c) Cyclic voltammograms of (a) NiMo/Ni(OH)$_2$/NiAl (electrodeposition for 1200 s) and (c) NiMo/Ni(OH)$_2$/NiAl (electrodeposition for 3600 s) in the double layer region (without Faradic process) at the different scan rates of 4, 6, 8, 10, 12, 14, 16 mV/s varying along the arrow direction. (b, d) Current density as a function of scan rate of (b) NiMo/Ni(OH)$_2$/NiAl-1200 and (d) NiMo/Ni(OH)$_2$/NiAl-3600.

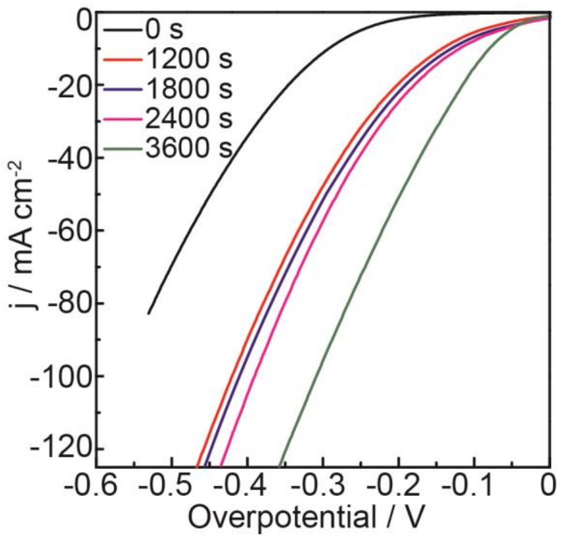

Figure S12. HER polarization curves of NiMo/$Ni(OH)_2$/NiAl prepared with different electrodeposition time.

Table S1. Comparison of OER performance of our NiFe/Ni(OH)$_2$/NiAl with the typical state-of-the-art electrocatalysts in 1 M KOH.

Catalysts	Overpotential at 10 mA cm^{-2} (mV)	Overpotential at 100/500 mA cm^{-2} (mV)	Reference
NiFe/Ni(OH)$_2$/ NiAl	256	315[a]/374[a]	**This work**
Ni(OH)$_2$/NiAl	289	--	
IrO$_2$	325	--	
NiFe/NiCo$_2$O$_4$/ NF	--	280[a]/325[a]	**Adv. Funct. Mater.** 2016, 26, 3515.
NiFe/NF	--	350/520	**Nat. Commun.** 2015, 6, 6616.
EG/Co$_{0.85}$SeNi Fe-LDH	--	260/--	**Energy Environ. Sci.** 2016, 9, 478.
NiFe-LDH/NF	240	460/--	**Science** 2014, 345, 6204.
Co-B$_i$ NS/G	290	380/--	**Angew. Chem. Int. Ed.** 2016, 55, 2488.
h-NiS$_x$	--	217/316	**Adv. Energy Mater.** 2016, 1502333.
Ni$_3$FeN-NPS	--	380/690	**Adv. Energy Mater.** 2016, 1502585.
NiCo$_2$S$_4$ NW/ NF	260	375/--	**Adv. Funct. Mater.** 2016, 26, 4661.
Mesporous Ni$_{60}$Fe$_{30}$Mn$_{10}$ metal/metal-oxide	200	--/360	**Energy Environ. Sci.** 2016, 9, 540.
FeOOH/CeO$_2$ HLNTS-NF	225	325/--	**Adv. Mater.** 2016, 28, 4698.
Core-ring NiCo$_2$O$_4$	--	315/--	**Nat. Mater.** 2011, 10, 780.

[a] The overpotential was given with iR-correction.

Table S2. Comparison of HER performance of NiMo/Ni(OH)$_2$/NiAl with the typical state-of-the-art electrocatalysts in 1 M KOH.

Catalysts	Overpotential at 10 mA cm^{-2} (mV)	Reference
NiMo/Ni(OH)$_2$/NiAl	78	**This work**
Ni(OH)$_2$/NiAl	292	
Pt/C	33	
NiSe/NF	96	**Angew. Chem. Int. Ed.** 2015, 54, 9351.
EG/Co$_{0.85}$Se/NiFe-LDH	110	**Energy Environ. Sci.** 2016, 9, 478.
Co-P films	94	**Angew. Chem. Int. Ed.** 2015, 54, 6251.
Ni$_5$P$_4$ on Nickel foil	150	**Angew. Chem. Int. Ed.** 2015, 54, 12361.
Ni$_3$FeN-NPS	158	**Adv. Energy Mater.** 2016, 1502585.
h-NiS$_x$	60	**Adv. Energy Mater.** 2016, 1502333.
CoO$_x$@CN	232	**J. Am. Chem. Soc.** 2015, 137, 2688.
N, P-G	700	**ACS Nano** 2014, 8, 5290.
MoO$_x$/Ni$_3$S$_2$/NF	106	**Adv. Funct. Mater.** 2016, 26, 4839.
Ni/NiO/CoSe$_2$	85	**Angew. Chem. Int. Ed.** 2013, 52, 8546.
CoP-CNT	124	**Angew. Chem. Int. Ed.** 2014, 126, 6828.
CoSe$_2$ NW/CC	130	**ACS Appl. Mater. Interface** 2015, 7, 3817.
NiMoN/CC	109	**Adv. Energy Mater.** 2016, 6, 160021.

Table S3. Comparison of overall water splitting performance of the typical state-of-the-art catalysts with ours in 1.0 M KOH in terms of the cell voltage at 10 mA cm^{-2}.

Catalysts	Cell voltage (V)	Reference
$NiFe/Ni(OH)_2/NiAl$ || $NiMo/Ni(OH)_2/NiAl$	1.59	**This work**
NiFe LDH/NF	1.70	**Science** 2014, 345, 1593.
$Ni(OH)_2/NF$	1.82	
$EG/Co_{0.85}Se/NiFe$-LDH	1.67	**Energy Environ. Sci.** 2016, 9, 478.
ONPPGC/OCC	1.66	**Energy Environ. Sci.** 2016, 9, 1210.
NiCoP/rGO	1.59	**Adv. Funct. Mater.** 2016, 26, 6785.
Ni_5P_4/NF	1.70	**Angew. Chem. Int. Ed.** 2015, 54, 13361.
Ni_3Se_2/CF	1.65	**Catal. Sci. Technol.** 2015, 5, 4954.
NiMo HNRS/TiM	1.64	**J. Mater. Chem. A** 2015, 3, 20056.
$NiCo_2S_4/CC$	1.68	**Nanoscale** 2015, 7, 15122.
Ni-P films	1.67	**ChemCatChem.** 2016, 8, 106.
